# A comparative study of effect of autograft compared with allograft anterior cruciate ligament reconstruction on expressions of *LOXs* and *MMPs*

**DOI:** 10.1042/BSR20160533

**Published:** 2017-04-28

**Authors:** Wei-Ming Wang, Xiao-Jun Ma, Shi-Bo Huang, Liu-Bao Ren, Yu-Peng Liu

**Affiliations:** Department of Orthopaedic Surgery, Affiliated Zhongshan Hospital of Dalian University, Dalian 116001, P.R. China

**Keywords:** Autograft, Allograft, Anterior cruciate ligament reconstruction, LOXs, MMPs

## Abstract

The present study aimed to compare the effect of autograft or allograft anterior cruciate ligament (ACL) reconstruction on the expressions of lipoxygenases (*LOXs*) and matrix metalloproteinases (*MMPs*) in a New Zealand white rabbit model. New Zealand white rabbits were divided randomly into control, sham, autograft and allograft groups. At the 4th and 8th week after operation, biomechanical testing was performed to measure the primary length, cross-sectional area, maximum tensile load and stiffness of ACL, and HE staining was used to observe cell morphology and fibre alignment of ACL. At the 2nd, 4th and 8th week after operation, quantitative real-time PCR (qRT-PCR), Western blotting and immunohistochemistry were applied to detect *LOXs* and *MMPs* expressions, and expressions of adenomatous polyposis coli (APC)/Wnt signalling pathway-related proteins. At the 4th and 8th week after operation, the maximum tensile load and stiffness were higher in the autograft group than in the allograft group, and the values at the 8th week were higher than those at the 4th week after operation. The fibroblast proliferation in the allograft group was more significant than that in the autograft group. Compared with the control group, *LOXs* and *MMPs* expressions and the positive expression rates of *LOXs* and *MMPs* proteins were elevated, and the values in the allograft group were higher than those in the autograft group at all time points. At 8th week after operation, compared with the autograft group, Wnt expression was higher and APC expression was lower in the allograft group. Autograft and allograft ACL reconstruction can promote *LOXs* and *MMPs* expressions by activating the APC/Wnt signalling pathway.

## Introduction

Anterior cruciate ligament (ACL), the most frequently injured ligament in the knee, had an occurrence of approximately 250000–300000 ACL injuries in the United States annually [1,2]. The ACL consists of the anteromedial and posterolateral bundles, which are responsible for the anterior stability of the knee and the control of tibial rotation respectively [3,4]. The ACL accounts for approximately 50% knee injuries caused by sports activities due to movements that cause extreme strain and rotation of the knee, direction change, immediate halting or outer impacts [5]. ACL reconstruction, as a kind of common orthopaedic procedure in surgery worldwide, is regarded as the standard surgical procedure for the treatment of ACL tear [6]. Occasionally combined with extra-articular plastic/augmentation, ACL reconstruction is carried out arthroscopically using autograft or allograft [7]. However, graft failure is an important clinical outcome after ACL reconstruction. Non-physiological loading in the knee can be caused by mismatched biomechanical properties of the tendon graft tissue to replace the native ligament tissue [8]. Accumulating studies have been carried out to explain the molecular mechanism for graft failure of the ACL for well healing after rupture. It is important to better understand the role of gene expression in normal and injured ACL for clinical guidance in patient management and development of new therapeutic options for ACL reconstruction [9].

Lipoxygenases (*LOXs*), a group of non-haem iron-containing dioxygenases, can promote the oxidation of polyunsaturated fatty acids (PUFAs) possessing a (Z,Z)-1,4-pentadiene structure to generate unsaturated fatty acid hydroperoxides [10]. *LOXs* are usually used to investigate the physiological functions of hydroxy- and hydroxyperoxy fatty acids [11]. Matrix metalloproteinases (*MMPs*) are a large family of zinc-containing endopeptidases, which can be grouped into collagenases, gelatinases, stromelysins, matrilysins, membrane-type *MMPs* and enamelysin [12]. *MMPs* are critical in a variety of processes associated with cell proliferation, invasion, differentiation, apoptosis, host defences and tissue remodelling such as morphogenesis, angiogenesis, arthritis, metastasis and tissue repair, and are involved in extracellular matrix (ECM) and basement membrane degradation [12,13]. *MMPs* have been reported to have affect on the growth and remodelling of neuronal cellular elements through proteolytic cleavage of ECM proteins in physiological plasticity, which generates soluble ligands binding to integrin as well as a free environment for reorganization [14]. To investigate the associations of autograft and allograft ACL reconstruction with the expressions of *LOXs* and *MMPs*, the present study was carried out in rabbit models undergoing ACL reconstruction with tendon autograft or allograft.

## Materials and methods

### Ethics statement

The experiments were conducted following the Guidance Suggestions for the Care and Use of Laboratory Animals issued by the Ministry of Science and Technology of the People’s Republic of China.

### Experimental animals

A total of 72 New Zealand white rabbits (The Experimental Animal Center of Dalian Medical University, Dalian, China) with a weight of 2.3–3.1 kg were selected. The observation was performed 2 weeks before operation and showed that the rabbits had normal diets and stable knee joint motion. Normal results were observed in the lateral stress test [15] and anterior and posterior drawer tests as well [16].

### Animal grouping and treatment

The 72 rabbits were randomly divided into control group, sham group, autograft group and allograft group, with 18 rabbits in each group. The rabbits in the control group were fed normally without any operation. In the sham group, the knee joint was opened to expose the ACL, and then the cut was sutured without other operation. In the autograft group and the allograft group, the rabbits were anaesthetized with pentobarbital injection through ear vein. After the semitendinosus was cut, folded into double strand and sutured with 3-0 tendon line, an interior peripatellar incision was made to expose the knee joint where the complete ACL was excised in the top and bottom dead centre. When the results of both Lachman testing [17] and anterior drawer test were positive, tibial and femoral bone tunnels with a 2-mm diameter were drilled and marked by the attachment point of the normal ACL. In the top and bottom dead centre, the traction suture was used to draw the semitendinosus tendon from the external aperture of bottom bone tunnel through the tibial and femoral bone tunnels. Then two parallel bone holes in the distal femur that formed the bone bridge were tied and fixed at 30° of knee flexion. The rabbits were implanted with the native ACL in the autograft group and the ACL excised from other animal models in the allograft group. After the Lachman test and anterior drawer test showed negative, the incision was sutured layer by layer. The affected knees were intramuscularly injected with penicillin for anti-infection without fixation on the first 3 days after operation. The experimental animals were killed in batches at the 2nd, 4th and 8th week after operation, with six rabbits in each group every time for the subsequent experiments.

### Biomechanical testing

Before biomechanical testing, the rabbits in the control group were killed for sample collection. At the 4th and 8th week after operation, the samples were collected in the sham, autograft and allograft groups, with six experimental animals in each group. The distal femur and proximal tibia (each approximately 3 cm) were reserved. All soft tissues, except the reconstructed ACL, were removed, sutured and stored at –80°C. Before operation, the samples were thawed, and the middle cross-sectional area of ligament was measured at a constant pressure (0.12 MPa, 120 s). The medial femoral condyle was removed, and then the femur and tibia of rabbits were fixed using barrel-shaped clamping apparatus and steel nails to make the tension pass through the axis of ligament. With measurement of the initial length of the reconstructed ACL, tensile testing was performed using MTS858 material testing system. When the tension reached 2.0 N, the length of ligament was measured by vernier caliper. Then the tensile test was done at 500 mm/min, and the testing system was used to draw the load-displacement curve. The absolute values were obtained for comparison.

### Haematoxylin-eosin staining

At the 4th and 8th week after operation, the ACL samples in all groups were observed by haematoxylin-eosin (HE) staining. The procedures were as follows: fixed in 10% neutral formaldehyde, the samples were paraffin embedded, sectioned and toasted. Sections were regularly dewaxed, rinsed, stained with haematoxylin for 50 s, differentiated with hydrochloric acid (1%) and ethanol (70%) for 20 s, stained with eosin for 6 min and dehydrated in gradient ethanol (80%, 95% I, 95% II, 100% I, 100% II) for 1 min, followed by transparency by xylene I and xylene II for 2 min respectively. After mounting with DPX rubber, the cell morphology, fibre alignment and cell status were observed under a light microscope.

### Quantitative real-time polymerase chain reaction

Total RNA was extracted using total RNA extraction kit (Beijing Tiandz Genetech Co., Ltd., Beijing, China) for animal cells at the 2nd, 4th and 8th week after operation, followed by reverse transcription to cDNA using reverse transcription-PCR kit (Hangzhou Bioer Technology Co., Ltd., Hangzhou, China). RT-PCR was performed to determine the mRNA expressions of *LOXs* (*LOX* and *LOX 1–4*) and *MMPs* (*MMP-1*, *MMP-2*, *MMP-3*) with SYBR Green (Shanghai Yingjie Chemical Co. Ltd., Shanghai, China) as the fluorescence signal. The primers for PCR reactions were designed by Premier 5.0 and Gene bank and the sequences were shown in Table 1. The amplified reaction system of quantitative real-time PCR (qRT-PCR) was 25 μl, including SYBR Green (12.5 μl), forward and reverse primers (1 μl each), cDNA (1 μl) and diethyl phosphorocyanidate (DEPC)-treated water (Shanghai Qifu Biotechnology Co., Ltd., Shanghai, China) (9.5 μl). The relative expressions of target genes were determined using glyceraldehyde-3-phosphate dehydrogenase (GAPDH) as the internal control, and the mRNA expression was calculated using the *C*_t_ values.

**Table 1:** The primer sequences of *LOXs* and *MMPs* for qRT-PCR

Gene	Sequence number	Primer sequence	Product/bp
GAPDH	NM_002046	F: 5′-GCACCGTCAAGGCTGAGAAC-3′	138
		R: 5′-TGGTGAAGACGCCAGTGGA-3′	
*LOX*	NM_002317	F: 5′-GCATACAGGGCAGATGTCAGA-3′	183
		R: 5′-TTGGCATCAAGCAGGTCATAG-3′	
*LOXL-1*	NM_005576	F: 5′-TGCCACCAGCATTACCACAG-3′	135
		R: 5′-GAGGTTGCCGAAGTCACAGG-3′	
*LOXL-2*	NM_002318	F: 5′-CTGCAAGTTCAATGCCGAGT-3′	149
		R: 5′-TCTCAACCAGCACCTCCACTC-3′	
*LOXL-3*	NM_032603	F: 5′-CAACAGGAGGTTTGAACGCTAC-3′	146
		R: 5′-GCTGACATGGGTTTCTTGGTAA-3′	
*LOXL-4*	NM_032211	F: 5′-TTCACCCACTACGACCTCCTCA-3′	155
		R: 5′-CAGCAGCCTACAGTCACTCCCT-3′	
*MMP-1*	NM_002421	F: 5′-GGCTGAAAGTGACTGGGAAACC-3′	170
		R: 5′-TGCTCTTGGCAAATCTGGCGTG-3′	
*MMP-2*	NM_005576	F: 5′-GTGACGGAAAGATGTGGTG-3′	179
		R: 5′-GGTGTAGGTGTAAATGGGTG-3′	
*MMP-3*	NM_002318	F: 5′-GACAAAGGATACAACAGGGAC-3′	122
		R: 5′-TGAGTGAGTGATAGAGTGGG-3′	

F, forward; R, reverse.

### Western blotting

The ACL samples of the killed rabbits in each group were harvested at the 2nd, 4th and 8th week after operation, and were then digested with trypsin and centrifuged to collect cells which were lysed to extract proteins. Proteins were separated using PAGE, transferred to PVDF membrane, and then blocked with Tris/HCl buffer saline solution (TBS) containing 5% skimmed milk. The membrane was incubated with the primary antibodies, including *LOX* (1:1000), Wnt1 (1:2000), adenomatous polyposis coli (APC) (1:2000) and β-actin (1:3000), at room temperature for 30 min and overnight at 4°C, then was incubated with the secondary antibody (1:10000) labelled by horseradish peroxidase (HRP) at room temperature for 30 min. Electrochemical luminescence was used for developing. The grey values of *LOXs* and *MMPs* protein bands to β-actin protein band represented the relative expressions of *LOXs* and *MMPs* proteins*.*

### Immunohistochemistry

The ACL samples of the killed rabbits in each group were harvested at the 2nd, 4th and 8th week after operation, and were stained by streptavidin-peroxidase-biotin (S-P), with positive sections as the positive control and PBS as the negative control. It was considered positive when the stained *LOXs* and *MMPs* had brownish yellow granules in the cytoplasm. A total of 10 high-power (×400) fields were selected randomly under light microscope and 200 cells were counted to calculate the percentage of positive cells and rate of positive expression.

### Statistical analysis

SPSS 19.0 software (SPSS Inc., Chicago, IL, U.S.A.) was used for statistical analysis. Differences in the means of each group were determined using ANOVA (F test). Student–Newman–Keuls (SNK) test was used for pairwise comparison of differences in the means of each group. Postoperative time-dependent variation of indicators was compared using independent samples *t* test. With α =0.05 and 95% confidence interval (CI), *P*<0.05 was considered as statistically significant.

## Results

### Biomechanical indexes of ACL in the control, sham, autograft and allograft groups

The biomechanical testing showed that at the 4th and 8th week after ACL reconstruction, there were no significant differences in the primary length, cross-sectional area, maximum tensile load and stiffness of reconstructed ACL between the sham group and the control group (all *P*>0.05). There were also no differences in the primary length and cross-sectional area of reconstructed ACL among the autograft group, the allograft group and the control group (all *P*>0.05), while the maximum tensile load and stiffness were significantly lower in the autograft and allograft groups than in the control and sham groups (all *P*<0.05). In the autograft group, the maximum tensile load and stiffness were remarkably higher in comparison with the allograft group (both *P*<0.05) and compared with the values at the 4th week, the maximum tensile load and stiffness were significantly elevated at the 8th week in the autograft and allograft groups (all *P*<0.05) (Table 2).

**Table 2:** The biomechanical indexes of reconstructed ACL in the control, sham, autograft and allograft groups at the 4th and 8th week after operation

Week	Index	Control group	Sham group	Autograft group	Allograft group
4	Primary length (mm)	11.81 ± 1.12	11.54 ± 1.06	9.83 ± 0.87	10.47 ± 1.02
	Cross-sectional area (mm^2^)	1.93 ± 0.17	2.04 ± 0.19	1.86 ± 0.15	1.90 ± 0.18
	Maximum tensile load (N)	45.54 ± 3.95	47.48 ± 4.38	36.26 ± 3.57*	30.71 ± 3.74*^†^
	Stiffness (N/mm)	28.18 ± 2.46	30.94 ± 2.93	17.52 ± 2.43*	10.48 ± 0.96*^†^
8	Primary length (mm)	11.85 ± 1.14	11.69 ± 1.01	10.64 ± 0.95	11.03 ± 1.04
	Cross-sectional area (mm^2^)	2.08 ± 0.21	1.94 ± 0.16	1.97 ± 0.18	2.03 ± 0.19
	Maximum tensile load (N)	48.29 ± 5.17	47.14 ± 4.22	41.94 ± 4.36*^‡^	35.28 ± 3.47*^†‡^
	Stiffness (N/mm)	26.96 ± 2.31	28.57 ± 2.71	22.03 ± 2.31*^‡^	15.69 ± 1.94*^†‡^

*, *P*<0.05 compared with the control group; ^†^, *P*<0.05 compared with the autograft group; ^‡^, *P*<0.05 compared with the indexes at the 4th week after operation.

### Postoperative histological changes of ACL in the control, sham, autograft and allograft groups

**Figure 1:**
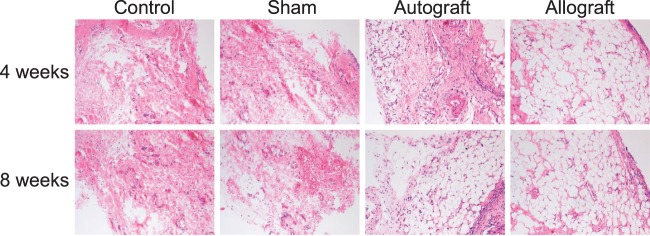
HE staining of ACL in the control, sham, autograft and allograft groups at the 4th and 8th week after operation. Notes:HE, hematoxylin-erosin, ACL, anterior cruciate ligament HE staining of ACL in the control, sham, autograft and allograft groups at the 4th and 8th week after operation.

HE staining indicated that there was no proliferation of synovial tissues and fibroblasts adjacent to the ACL in the control and sham groups. In the autograft group, at the 4th week after operation, obvious fibroblast proliferation was observed in the transplantation site of grafted ACL and significant increase in fibrocytes was found in the central region of grafted ACL, without broken collagen. At the 8th week, the ligament–bone interface healed well, the fibroblast proliferation around the grafted ACL decreased compared with that at the 4th week, and the collagen had matured in a regular orientation. In the allograft group, at the 4th week, the synovial cells adjacent to the grafted ACL increased significantly, the fibroblasts were distributed irregularly and the fibrocytes in the centre of grafted ACL reduced significantly. At the 8th week, the fibroblasts still increased significantly, the ligament–bone interface healed well, the collagen distributed regularly, differentiated and matured completely, and the fibroblasts in the centre of grafted ACL increased compared with those at the 4th week (Figure 1).

### The mRNA expressions of *LOXs* and *MMPs* in the control, sham, autograft and allograft groups after operation

**Figure 2:**
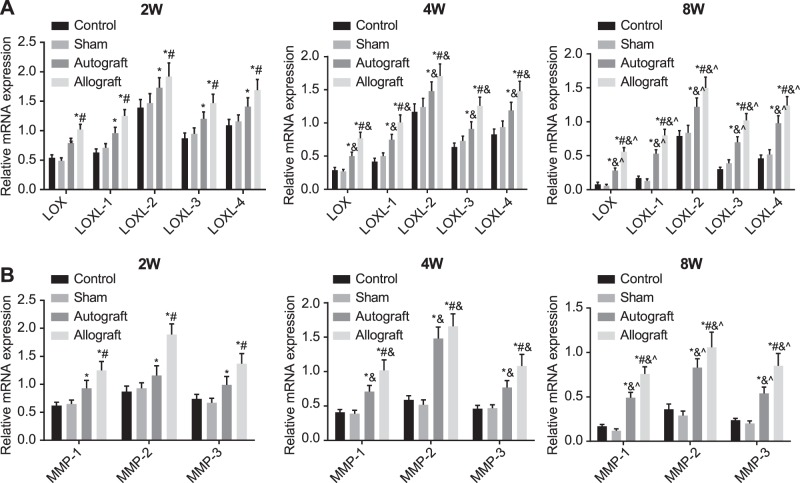
The mRNA expression of LOXs(A)and MMPs(B)in the control, sham, autograft and allograft groups at the 2nd, 4th and 8th week after operation detected by qRT-PCR The mRNA expressions of *LOXs* (**A**) and *MMPs* (**B**) in the control, sham, autograft and allograft groups at the 2nd, 4th and 8th week after operation detected by qRT-PCR. *, *P*<0.05 compared with the control group; ^#^, *P*<0.05 compared with the autograft group; ^&^, *P*<0.05 compared with the indexes at the 2nd week after operation; ^∧^, *P*<0.05 compared with the indexes at the 4th week after operation.

Compared with the control group, the mRNA expressions of *LOXs* and *MMPs* were elevated in the autograft and allograft groups at the 2nd, 4th and 8th week after operation (all *P*<0.05), while there were no significant differences in the mRNA expressions of *LOXs* and *MMPs* between the control group and the sham group (all *P*>0.05). The mRNA expressions of *LOXs* and *MMPs* in the allograft group were increased compared with the autograft group at all time points after operation (all *P*<0.05). The mRNA expressions of *LOXs* family genes in the autograft and allograft groups reached the highest point at the 2nd week after operation, after which the expressions decreased gradually and the mRNA expression rank was *LOXL-2* > *LOXL-4* > *LOXL-3* > *LOXL-1* > *LOX* (Figure 2A). Among *MMPs* family genes, the mRNA expressions of *MMP-1* and *MMP-3* in the autograft and allograft groups reached the maximum at the 2nd week after operation, while the mRNA expression of *MMP-2* reached the maximum at the 4th week after operation in the autograft group and reached the maximum at the 2nd week after operation in the allograft group, and the mRNA expression of *MMP-2* in the allograft group was significantly higher than that in the autograft group (*P*<0.05) (Figure 2B).

### The expressions of *LOXs* and *MMPs* proteins and the APC/Wnt signalling pathway-related proteins in the control, sham, autograft and allograft groups after operation

**Figure 3:**
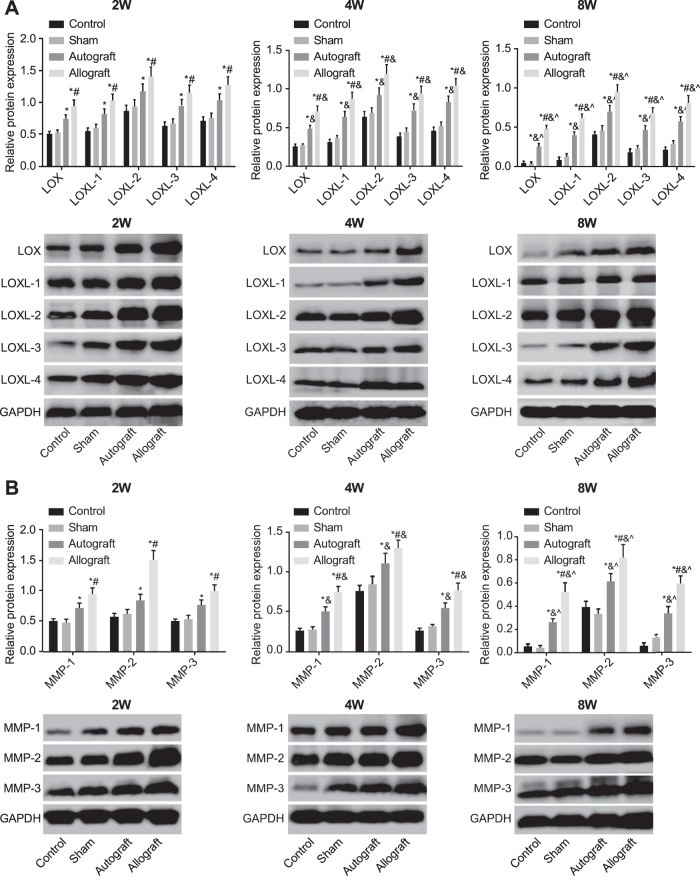
The protein expressions of LOXs(A) and MMPs(B) in the control, sham, autograft and allograft groups at the 2nd, 4th and 8th week after operation detected by Western blotting The protein expressions of *LOXs* (**A**) and *MMPs* (**B**) in the control, sham, autograft and allograft groups at the 2nd, 4th and 8th week after operation detected by Western blotting. *, *P*<0.05 compared with the control group; ^#^, *P*<0.05 compared with the autograft group; ^&^, *P*<0.05 compared with the indexes at the 2nd week after operation; ^∧^, *P*<0.05 compared with the indexes at the 4th week after operation.

There were no significant differences in relative protein expressions of *LOXs* and *MMPs* between the sham group and the control group at each time period after operation (all *P*>0.05). The protein expressions of *LOXs* and *MMPs* were up-regulated in the autograft and allograft groups at the 2nd, 4th and 8th week after operation (all *P*<0.05), and the protein expressions of *LOXs* and *MMPs* in the allograft group were higher than those in the autograft group at each time period after operation (all *P*<0.05). The protein expressions of *LOXs* in the autograft and allograft groups reached the maximum at the 2nd week after operation, and then the expressions decreased gradually (all *P*<0.05). The protein expression rank of *LOXs* family genes was *LOXL-2* > *LOXL-4* > *LOXL-3* > *LOXL-1* > *LOX*, which was consistent with the results of qRT-PCR (Figure 3A). The protein expressions of *MMP-1* and *MMP-3* reached the maximum at the 2nd week in the autograft and allograft groups, and the expression in the allograft group was higher than that in the autograft group (both *P*<0.05). In the autograft group, the protein expression of *MMP-2* reached the maximum at the 4th week, while the expression reached the maximum at the 2nd week in the allograft group (Figure 3B).

**Figure 4:**
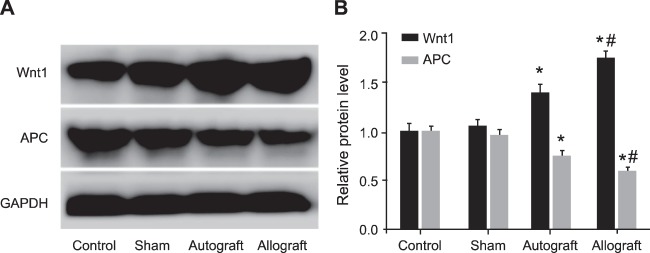
The expression of the APC/Wnt signaling pathway-related proteins in the control, sham, autograft and allograft groups at the 8th week after operation detected by Western blotting The expressions of the APC/Wnt signalling pathway-related proteins in the control, sham, autograft and allograft groups at the 8th week after operation detected by Western blotting. *, *P*<0.05 compared with the control group; ^#^, *P*<0.05 compared with the autograft group.

The expressions of the APC/Wnt signalling pathway-related proteins at the 8th week after operation were displayed in Figure 4. Compared with the control group, the Wnt protein expression in the autograft and allograft groups were significantly increased, while the APC expression in the two groups significantly reduced (all *P*<0.05). Compared with the autograft group, the Wnt protein expression was elevated and the APC expression was decreased in the allograft group (both *P*<0.05).

### Positive expression rate of *LOXs* and *MMPs* proteins in the control, sham, autograft and allograft groups after operation

The immunohistochemistry suggested that at the 2nd, 4th and 8th week after operation, there were no significant differences in the positive expression rates of *LOXs* and *MMPs* proteins between the control and sham groups (all *P*>0.05). In the autograft and allograft groups, the positive expression rates of *LOXs* and *MMPs* proteins were significantly higher than those in the control group, and those in the allograft group were higher in comparison with the autograft group (all *P*<0.05). In the autograft and allograft groups, the positive expression rates of *LOXs*, *MMP-1* and *MMP-3* proteins reached the maximum at the 2nd week and gradually reduced (all *P*<0.05). In the autograft group, the positive expression rate of *MMP-2* protein reached the maximum at the 4th week, while the positive expression rate of *MMP-2* protein reached the maximum at the 2nd week in the allograft group (Tables 3 and 4).

**Table 3:** Positive expression rate of *LOXs* protein in the control, sham, autograft and allograft groups at the 2nd, 4th and 8th week after operation

Week	Gene	Control group	Sham group	Autograft group	Allograft group
2	*LOX*	20.95 ± 1.84	21.17 ± 2.32	31.38 ± 3.56*	42.73 ± 4.17*^†^
	*LOXL-1*	23.12 ± 2.67	23.39 ± 2.45	33.94 ± 3.82*	44.26 ± 4.26*^†^
	*LOXL-2*	37.28 ± 4.15	35.53 ± 3.72	47.14 ± 4.91*	56.45 ± 5.42*^†^
	*LOXL-3*	26.35 ± 2.53	28.14 ± 3.18	38.22 ± 4.51*	48.87 ± 5.27*^†^
	*LOXL-4*	32.61 ± 3.39	31.48 ± 3.23	42.07 ± 4.95*	52.56 ± 5.86*^†^
4	*LOX*	10.25 ± 1.49	11.12 ± 1.73	21.34 ± 3.12*^‡^	32.19 ± 3.65*^†^^‡^
	*LOXL-1*	12.53 ± 2.26	13.04 ± 1.94	23.16 ± 3.06*^‡^	33.47 ± 4.13*^†^^‡^
	*LOXL-2*	26.34 ± 2.85	25.18 ± 3.32	35.23 ± 4.28*^‡^	45.52 ± 4.96*^†^^‡^
	*LOXL-3*	15.73 ± 2.47	15.52 ± 2.53	26.21 ± 3.71*^‡^	37.94 ± 4.34*^†^^‡^
	*LOXL-4*	21.26 ± 2.89	21.05 ± 3.14	31.29 ± 3.82*^‡^	41.87 ± 4.36*^†^^‡^
8	*LOX*	0.24 ± 0.03	1.02 ± 0.14	11.33 ± 2.18*^‡§^	22.12 ± 2.36*^†^^‡§^
	*LOXL-1*	2.35 ± 0.39	2.39 ± 0.52	12.42 ± 2.39*^‡§^	22.86 ± 3.12*^†‡§^
	*LOXL-2*	13.23 ± 1.35	13.54 ± 1.83	24.38 ± 3.72*^‡§^	35.15 ± 4.16*^†^^‡§^
	*LOXL-3*	5.41 ± 0.67	5.26 ± 0.89	16.15 ± 1.63*^‡§^	27.29 ± 3.43*^†‡§^
	*LOXL-4*	10.14 ± 1.45	9.74 ± 1.32	20.37 ± 2.81*^‡§^	31.58 ± 4.13*^†^^‡§^

*, *P*<0.05 compared with the control group; ^†^, *P*<0.05 compared with the autograft group; ^‡^, *P*<0.05 compared with the indexes at the 2nd week after operation; ^§^, *P*<0.05 compared with the indexes at the 4th week after operation.

**Table 4:** Positive expression rate of *MMPs* protein in the control, sham, autograft and allograft groups at the 2nd, 4th and 8th week after operation

Week	Gene	Control group	Sham group	Autograft group	Allograft group
2	*MMP-1*	23.17 ± 2.64	24.09 ± 2.02	36.26 ± 3.13*	47.72 ± 4.65*^†^
	*MMP-2*	28.54 ± 1.43	30.37 ± 3.56	45.52 ± 4.18*	83.36 ± 8.75*^†^
	*MMP-3*	24.23 ± 2.13	25.91 ± 1.95	39.45 ± 3.72*	51.62 ± 4.41*^†^
4	*MMP-1*	12.82 ± 1.25	13.23 ± 1.83	25.12 ± 2.66*^‡^	34.98 ± 3.47*^†‡^
	*MMP-2*	15.21 ± 1.95	18.14 ± 1.72	58.23 ± 6.89*^‡^	69.74 ± 7.32*^†^^‡^
	*MMP-3*	13.08 ± 1.86	14.86 ± 1.89	27.79 ± 1.94*^‡^	38.68 ± 2.31*^†‡^
8	*MMP-1*	2.57 ± 0.28	2.96 ± 0.92	13.39 ± 1.54*^‡§^	24.43 ± 2.23*^†‡§^
	*MMP-2*	3.76 ± 0.27	5.53 ± 0.86	34.46 ± 4.04*^‡§^	57.12 ± 6.75*^†^^‡^^§^
	*MMP-3*	2.84 ± 0.34	3.64 ± 0.91	15.23 ± 1.62*^‡^^§^	26.35 ± 2.18*^†^^‡^^§^

*, *P*<0.05 compared with the control group; ^†^, *P*<0.05 compared with the autograft group; ^‡^, *P*<0.05 compared with the indexes at the 2nd week after operation; ^§^, *P*<0.05 compared with the indexes at the 4th week after operation.

## Discussion

ACL reconstruction is required for athletes because destructive ACL seldom regain their original strength in the natural course of complete tear [18]. The injured ACL is regarded to display a poor healing response with few successful attempts at surgical repair [19]. ACL reconstruction with bioactive synthetic grafts works not only as a prosthesis but also as a scaffold on to which natural tissue grows [20]. The current graft options used for ACL reconstruction are commonly autografts or allografts. Increasing studies have reported a time-dependent change of gene expression after ACL reconstruction surgery during the graft remodelling process [21,22]. Therefore, our study investigated the association of autograft and allograft ACL reconstruction with the expressions of *LOXs* and *MMPs*.

Our data suggested that the mRNA and protein expressions of *LOXs* and *MMPs*, and the fibroblast proliferation were increased in the allograft group compared with the autograft group, indicating that increased expressions of *LOXs* and *MMPs* might predict poor prognosis of ACL reconstruction. As the major mediators of cartilage destruction, *MMPs*, as a family of zinc-dependent proteases, are active in degrading ECM proteins, maintaining homoeostasis of extracellular microenvironments and breaking down the components of ECM [23,24]. *MMP-2*, a kind of overexpressed *MMP* in the synovium, is anchored on cell surfaces following secretion to target their substrates in the surrounding cellular environment and highly expressed in synoviocytes, endothelial cells, infiltrating monocytes and macrophages, especially in the pannus [25]. *MMPs* are involved in a wide variety of biological processes and pathologies, such as cancer and arthritis, and inflammatory cytokines can cause an up-regulation of *MMPs* in normal chondrocytes [26]. *MMPs* expression increased in the ACL itself after ACL injury [27]. Therefore, the graft bone tunnel healing could be improved by inhibiting *MMPs* expression [28]. The increase in *MMP* expression in the ligament is consistent with prior report that levels of *MMP-1* and *MMP-3* in the knee were significantly higher, suggesting that increased levels of both enzymes in the knee indicate enhanced severity of synovial inflammation and also accelerated activity of joint destruction [29]. Based on the past study, the increased expression of *MMP-3* under tensile strain may be correlated with the formation of a new ligament around the grafted scaffold by breaking down the provisional tissue and mediating the proteolysis of certain substrates [30]. Another study also evidenced that Wnt3a protein could promote the mRNA expression of *MMP-2* in cell invasion [31]. More importantly, Wu et al. [32] demonstrated that the expression of *MMPs* was induced by Wnts by acting as the transcriptional targets of Wnt signalling, which was consistent with our result that the expressions of *MMPs* and Wnt protein were higher in the allograft group than in the autograft group. APC is a crucial factor negatively regulating Wnt signalling in the process of tumor formation and cell development, which forms the destruction complex through the combination with the kinases CK1 and GSK3 and the scaffold Axin to target β-catenin [33]. Thereby the reduced APC expression would result in increased expression of Wnt, which was supported by our results that in the allograft group, the APC expression was lower than the autograft group. *LOXs* play central roles in lipid peroxidation under biotic and abiotic stresses, and the oxygenated lipids initiate activation of cellular signalling mechanisms via specific receptors on cell surfaces or further metabolization into potent lipid mediators [34,35]. In addition, *LOXs* are involved in more than three different types of catalytic reactions, including oxidation of the lipid double plus (peroxidase reaction), a secondary lipid peroxide conversion (reaction of hydrogen peroxidase) and formation of epoxy leucotrienes (leukotriene synthesis reaction) [36]. It has been recently reported that the gene expression of *LOX* is related to the total amount of enzymatic cross-linking and cross-linking patterns, which gives evidence that an increase in *LOX* mRNA expression is indicative of increased amounts of functional protein [37]. Therefore, it can be concluded that elevated expressions of *LOXs* and *MMPs* are related to worse healing of reconstructed ACL, which was consistent with our results.

Besides, our results found that the maximum tensile load and stiffness in the autograft group were significantly higher in comparison with the allograft group, suggesting that autograft in ACL reconstruction has better clinical effects than allograft. Decreased expression of *LOX* in human ACL cells could contribute to decreased stiffness of developing tendons and ligament strength and increased laxity in the knee [38]. Furthermore, down-regulation of *MMP* inhibits graft degradation, enhances ACL grafts incorporation, causes a remarkably higher load to failure and promotes more fibres at the tendon–bone interface [23]. It has been found that allograft has higher rate of clinical failure than autograft, implicating that autograft is superior to allograft [6,39–41]. In addition, another study also indicated that autograft achieved better clinical outcomes in terms of clinical failure compared with allograft [42].

Taken together, our study has provided evidence that high expressions of *LOXs* and *MMPs* might be related with poor prognosis of ACL reconstruction, and the autograft had a better effect in ACL reconstruction than the allograft. Our analysis also had the limitation that there were only comparisons between the autograft and allograft in ACL reconstruction. Therefore, the differences among the autograft, irradiated allograft and non-irradiated allograft need to be further investigated. But the present study provides evidence that *LOXs* and *MMPs* could be used as therapeutic biomarkers in the future treatment of ACL injury in order to improve the prognosis of ACL reconstruction.

## Abbreviations

ACL: anterior cruciate ligament
APC: adenomatous polyposis coli
CR1: casein kinase 1
DPX: dibutyl phthalate xylene
ECM: extracellular matrix
GAPDH: glyceraldehyde-3-phosphate dehydrogenase
GSK3: glycogen synthase kinase-3
HE: haematoxylin-eosin
LOX: lipoxygenase
LOXL: lysine oxidase-like
MMP: matrix metalloproteinase
MTS858: material testing machine (MTS)858
qRT-PCR: quantitative real-time PCR
RT-PCR: real-time polymerase chain reaction
Wnt: wingless-int
